# Evaluation of Kato-Katz and multiplex quantitative polymerase chain reaction performance for clinical helminth infections in Thailand using a latent class analysis

**DOI:** 10.1098/rstb.2022.0281

**Published:** 2023-08-21

**Authors:** Chawarat Rotejanaprasert, Pavadee Chuaicharoen, Joaquin M. Prada, Thanawadee Thantithaveewat, Poom Adisakwattana, Wirichada Pan-ngum

**Affiliations:** 1 Department of Tropical Hygiene, Faculty of Tropical Medicine, Mahidol University, Bangkok, Thailand; 2 Mahidol-Oxford Tropical Medicine Research Unit (MORU), Faculty of Tropical Medicine, Mahidol University, Bangkok, Thailand; 3 Department of Helminthology, Faculty of Tropical Medicine, Mahidol University, Bangkok, Thailand; 4 Faculty of Health and Medical Sciences, School of Veterinary Medicine, University of Surrey, Guildford, UK; 5 Bureau of General Communicable Diseases, Ministry of Public Health, Nonthaburi, Thailand

**Keywords:** health and disease and epidemiology, helminth, elimination, Kato-Katz, qPCR, latent class analysis

## Abstract

Using an appropriate diagnostic tool is essential to soil-transmitted helminth control and elimination efforts. Kato-Katz (KK) is the most commonly used diagnostic, but recently other tools, such as real-time quantitative polymerase chain reaction (multiplex qPCR), are starting to be employed more. Here, we evaluated the performance of these two diagnostic tools for five helminth species in Thailand. In the absence of a gold standard, diagnostic performance can be evaluated using latent class analysis. Our results suggest that in moderate to high prevalence settings above 2% multiplex qPCR could be more sensitive than KK, this was particularly apparent for *Opisthorchis viverrini* in the northeastern provinces. However, for low prevalence, both diagnostics suffered from low sensitivity. Specificity of both diagnostics was estimated to be high (above 70%) across all settings. For some specific helminth infection such as *O. viverrini*, multiplex qPCR is still a preferable choice of diagnostic test. KK performed equally well in detecting *Ascaris lumbricoides* and *Taenia solium* when the prevalence is moderate to high (above 2%). Neither test performed well when the prevalence of infection is low (below 2%), and certainly in the case for hookworm and *Trichuris trichiura*. Combination of two or more diagnostic tests can improve the performance although the cost would be high. Development of new methods for helminth surveillance at the pre-elimination phase is therefore very important.

This article is part of the theme issue ‘Challenges and opportunities in the fight against neglected tropical diseases: a decade from the London Declaration on NTDs’.

## 1 Introduction

Helminthiasis is a parasitic disease caused by infection with one or more worm (helminth) species, with the major helminthiasis, including soil-transmitted helminthiases (STH), e.g. *Ascaris lumbricoides* (roundworm), *Trichuris trichiura* (*Tr. trichiura* or *whipworm*) and *Ancylostoma duodenale* or *Necator americanus* (hookworm), food-borne trematodiases, e.g. *Opisthorchis viverrini* and taeniasis and cysticercosis, e.g. *Taenia solium* (*Taenia* spp.), classed as neglected tropical diseases by the World Health Organisation (WHO) [1]. Common morbidities of these STH include malnutrition and cognitive process impairment, with more severe forms including intestinal obstruction and rectal prolapse, which require surgical procedures [2]. The WHO estimated that 800 million people were infected with at least one species of STH, and the global disease burden was estimated to be more than 3 million disability-adjusted life years lost [3]. *Opisthorchis viverrini* infection is a significant risk factor for cholangiocarcinoma and several hepatobiliary diseases including cholangitis, obstructive jaundice, and hepatomegaly. *Opisthorchis viverrini* is mainly found in Southeast Asian countries bordering the Lower Mekong River, including Thailand, Laos, Cambodia and Vietnam [4]. *Taenia solium* infection induces taeniasis, which is normally asymptomatic, but may lead to neurocysticercosis owing to cysticerci development in the brain, causing chronic headaches, epilepsy, intracranial hypertension and other neurological symptoms [1,5].

In Thailand, the national survey conducted by the Ministry of Public Health in 2009 and 2019 showed that helminthiases prevalence had decreased from 18.1% to 9.79% [6,7]. The two most prevalent helminthiases observed were hookworm and *O. viverrini,* however, the intensity of infection was light on average [7]. Hookworm infection is predominant in the southern regions of Thailand, while *O. viverrini* is mainly found in the northeastern regions [8–10]. *Ascaris lumbricoides*, *Tr. trichiura* and *Taenia* spp. were reported in Chiang Mai, the north of Thailand [11,12].

The egg-based Kato-Katz (KK) technique is the most frequently used method to diagnose helminth infections, especially for STH in Southeast Asia and Thailand [8–11,13]. This technique is recommended by WHO for diagnosis and monitoring large-scale treatment programmes implemented for STH infection [14], as it is a relatively low-cost, standardized tool that is easy to perform in low-resource field settings [15]. KK is also quantitative, as the number of eggs counted can be used as a proxy for the intensity of infection, however, it might not be suitable for monitoring the control programmes where prevalence is low [16]. A significant limitation of this technique is its suboptimal accuracy, especially in low-transmission areas or during an early phase of elimination in which both the worm and the egg counts are usually low, and thus can be easily missed [17–19]. Moreover, KK was shown to be limited in its ability to detect liver fluke eggs including *O. viverrini* and *Clonorchis sinensis* in low-intensity infection, and it is common for multiple samples to be taken from each individual to improve diagnostic performance. For instance, a previous study reported the prevalence of clonorchiasis by a single smear underestimated over 40% compared to that determined by six smears from two stool samples [19]. Another limitation of KK is that hookworm eggs will disappear if the slide is not read within 30–60 min [20]. Furthermore, morphologically, *O. viverrini* is very similar to minute intestinal trematodes, resulting in misclassification when using the KK technique [21]. This can lead to unreliable estimations of prevalence, which will in turn impact decisions made for control and intervention programme activities. With limited spatial and temporal surveillance data, using KK alone might not be adequate to guide the national public health strategies for helminth control elimination [8,22]. Therefore, exploring alternative diagnostic tools is needed for a precise assessment of the real burden and intensity of these parasitic infections [23].

As mentioned above, the low sensitivity of KK, particularly at low intensities of infection, will result in the underestimation of true prevalence, and thus the improvement of diagnostic sensitivity is key to appropriately assess elimination [24]. Potentially more sensitive molecular methods such as multiplex quantitative polymerase chain reaction (qPCR) could be used 2 instead, or alternative sampling strategies for KK including multiple slides from multiple stools, or the pooling of samples, can also be used to increase sensitivity [25–27]. In Thailand, reliable and practical diagnostic tests, with appropriate sampling strategies, are urgently required to evaluate ongoing control programmes and surveillance systems for the helminth elimination phase.

Multiplex and multi-parallel qPCR methods have been developed and applied to detect STH DNA in stools [28,29]. These assays use only a single stool sample to detect multiple helminths simultaneously. Studies have shown that qPCR is more sensitive than a single/double KK slide, estimating high prevalence for STH in several settings [23,27,30–32]. Additionally, a study suggested that qPCR may also be used to estimate infection intensity almost four times more precisely than KK for the egg intensity of *As*. *lumbricoides* [33]. Such improvement could enhance the statistical power to inform whether helminth prevalence meets the elimination threshold of less than 2% for moderate and heavy intensity infections [1].

In general, to evaluate diagnostic accuracy (sensitivity, specificity, etc.), the results of the diagnostic are compared to a reference ‘gold’ standard in, for example, a case-control set-up. However, for these STH there is no ‘gold standard’, however, misclassification could be reduced through the combination of more than one diagnostic [17]. Multiple tests may, for example, be used in expert panels in which a group of clinicians reaches consensus based on the available test results of patients [18,34–37]. Results from multiple tests can also be combined through fixed rules to create a composite reference standard [38]. Alternatively, statistical approaches such as latent class analysis (LCA) can be used to evaluate multiple diagnostics in the absence of a ‘gold standard’. LCA allow simultaneous estimation of both the unknown true prevalence of infection, as well as these diagnostic characteristics such as sensitivity and specificity. This approach has been previously applied to the evaluation of imperfect diagnostic tests for vector-borne diseases [39,40] and other neglected tropical diseases [41–45], as well as evaluating STH diagnostic methods and meta-analysis [18,26,46,47].

In this study, we used LCA to evaluate the diagnostic performance of KK and multiplex qPCR in Thai vulnerable populations along the neighbouring-country borders of Myanmar, Laos and Cambodia. This included three different settings of varying disease prevalence and intensity of infection. Moreover, we considered these two diagnostics in the context of their advantages and disadvantages in implementation (table 1). The aim is to provide recommendations as to what diagnostic tool (or combination of tools) is more appropriate in these different settings. This will in turn inform the development of appropriate guidelines for the elimination of STH in Thailand, supporting control and elimination strategies, impact assessment of these programmes and further planning.

## 2 Methods

### (a) Study area, population and sample collection

Epidemiological data of faecal helminth egg positive detections with KK and multiplex qPCR in villagers living near three different Thai border regions: (i) Thailand–Myanmar border: the Mae Song Sub-District, Thasongyang District in Tak Province; (ii) Thailand-Lao border: the Kham Khuean Kaeo Sub-District of Sirindhorn District in Ubon Ratchathani Province; and (iii) Thailand–Cambodia region: the Phran Sub-District of Khun Han District in Sisaket Province were obtained from a recent study [23].

Briefly, in the study by Adisakwattana *et al*. [23], 567 faecal samples were collected between December 2017 and February 2018, and analysed by KK and multiplex qPCR analysis immediately after the end of sample collection. One hundred and sixty seven faecal samples from Tak, 200 samples from Ubon Ratchathani and 200 samples from Sisaket. KK analysis was conducted on site where two slides for each sample were prepared and analysed independently by two trained microscopists. All samples were anonymized and analysed blinded. Meanwhile, parallel faecal samples were stored in 80% (v/v) ethanol for transport to Bangkok for multiplex qPCR analysis. A sample was considered positive if there was at least one egg on a KK slide or quantification cycle (Cq) score < 37 by multiplex qPCR, based on the maximum Cq value for detection of all helminthic infection in Adisakwattana *et al.* [23].

### (b) Preparation and processing of samples for the multiplex quantitative polymerase chain reactionPCR

Firstly, faecal DNA was isolated using a QIAamp Fast DNA Stool Mini Kit (Qiagen GmbH, Hilden Germany). The method was designed to detect three different helminth species simultaneously as follows: assay 1: *As. lumbricoides*, *Strongyloides stercoralis*, *Tr. trichiura*; assay 2: *Ancylostoma* spp., *N. americanus*, *O. viverrini*; assay 3: *Ta. solium*, *Taenia saginata*, *Schistosoma japonicum*/*Schistosoma mekongi*. Multiplex qPCR was performed in duplicate in a final volume of 20 µl by mixing 1 µl of faecal DNA (100 ng µl^−1^) with 10 µl of iQ Multiplex Powermix (Bio-Rad Laboratories Inc., Hercules, CA, USA) and 200 nM each of forward (Fw) and reverse (Rv) primers in addition to 100 nM of the appropriate TaqMan probe. Amplification was performed using CFX96 Real-Time PCR System (Bio-Rad Laboratories, Hercules, CA) with pre-incubation at 95°C for 2 min, followed by 40 cycles of 95°C for 10 s and 60°C for 30 s. All positive control samples and a subset of clinical samples were checked for assay specificity by sequencing amplicons to confirm the identity of the helminth species detected. The detail was published in the electronic supplementary material, table S1 of the Additional file 1 in Adisakwattana *et al.* [23].

### (c) Evaluation of diagnostic performance by latent class analysis

True helminthic infection status of a subject is referred to as an unobserved (latent) variable, with two mutually exclusive possible states: ‘infection’ and ‘non-infection’. This unobserved variable controls the test probability (positive or negative) to a number of diagnostic tests. Knowledge of the accuracy of the test is represented by the sensitivity and specificity and some prior knowledge of disease prevalence in the populations of interest [52]. The model was conducted under the conditional independent assumption, similarly to some previous studies where two tests were assumed to be biologically unrelated [39,53–57]. For more information on LCA, the methods in the work of van Smeden *et al*. [58], Collins & Huynh [56] and Collins & Albert [59] can be reviewed.

[52] To conduct the simulations, the models were computed using OpenBUGS software [52,56]. Markov chain Monte Carlo inference was initialized using two chains with different values. Each chain comprised 100 000 samples, with the first 50 000 being discarded as the burn-in period. The chain convergence was assessed by visualization of trace plots of selected parameters and the Gelman–Rubin diagnostic. The posterior distribution of the sensitivity, specificity and prevalence were reported as the mean and the corresponding 95% posterior Bayesian credible intervals (BCI). Uninformative (flat) priors were used throughout, all model details are described in the electronic supplementary material, File 1: text S1 and S2.

## 3 Results

A total of 567 faecal samples from Thailand border regions, Tak province (Thailand–Myanmar, Ubon Ratchathani (Thailand-Lao), and Sisaket (Thailand–Cambodia), were analysed by KK and multiplex qPCR methods. Five species of helminth infections including (i) *O. viverrini*, (ii) *As. lumbricoides,* (iii) hookworm, (iv) *Taenia* spp., and (v) *Tr. trichiura* were found in the three border areas of Thailand (table 2). Across the two diagnostics, the Bayesian LCA estimated the median prevalence and 95% BCI for all species, which covered, as expected, the values previously reported [23]. Hookworm and *As. lumbricoides* were the two predominant helminths in the western border in Tak province, albeit at an estimated moderate-low prevalence (11–15% and 7–8% respective). *Opisthorchis viverrini* is, on the other hand, the most prevalent in the other two regions: in Ubon Ratchathani province (the northeastern border) 12–27% and in Sisaket 13–22% [23]. The estimated prevalence of *O. viverrini* varied significantly between the two diagnostics, with multiplex qPCR consistently estimating higher prevalence than KK.

Estimates of sensitivity of the two diagnostic tests for each parasite species varied across the different settings, with multiplex qPCR being more sensitive on average in most instances than KK. For example, for *O. viverrini* the average sensitivity was around 89–94% for multiplex qPCR compared to the average 60–73% estimated sensitivity with KK when prevalence was moderate (approx. 20%). However, across these three settings, the prevalence of some of the parasites detected was very low (1–4%), such as for *O. viverrini* or *Taenia spp.* in Tak province, which meant that the uncertainty in the estimation of sensitivity of the two diagnostics is very high (table 2). Specificity was generally above 70% for both tests, except for hookworm where the tests failed to perform, see more details in the electronic supplementary material, File 2: table S1.

## 4 Discussion

We evaluated the accuracy of two diagnostic tests, KK and multiplex qPCR, for typical helminth infections including *O. viverrini*, *As. lumbricoides*, hookworm, *Taenia* spp. and *Tr. trichiura* in three settings in Thailand using a Bayesian LCA without assuming a ‘gold standard’. The study sites considered represented three different levels of socio-economic status. Tak province, in the western border of Thai–Myanmar, is known to have a low socio-economic status, with a mixed population of Thai and Karen refugees. Various species of helminthic infections are present in the area, predominately hookworm and *As. lumbricoides*, which are common in these areas with poor access to water, sanitation and hygiene. Sisaket, bordering Cambodia, is always in the list of 10 poorest provinces in the northeast region of Thailand. Similarly to Tak, hookworm is also prevalent in the area. In addition, *O. viverrini* was commonly found in the northeast region of Thailand including Sisaket and Ubon Ratchathani. *Opisthorchis viverrini* is a food-borne trematode fluke caused by consumption of raw or undercooked fish, a long tradition of eating and sharing food in the region and across countries in the Indochinese peninsula (Thailand, Laos, Myanmar and Cambodia [60]). *Trichuris trichiura* and *Taenia* spp. were both detected in Ubon Ratchathani, one of four big cities in the region neighbouring the Mekong River, the border between Thailand and Laos PDR, and in Tak province, but not in Sisaket province.

Overall, multiplex qPCR is shown to be a suitable test for detecting *O. viverrini* in our study. While this was particularly apparent for the moderate prevalence (approx. 20%) settings 4 of Ubon Ratchathani and Sisaket, in Tak province, where prevalence was very low, it was not possible to confirm owing to the large uncertainty [23,61]. Both KK and multiplex qPCR, are able to detect *As. lumbricoides* and *Taenia* spp. with a high estimated average sensitivity (approx. 88%) if the prevalence is not too low (above 3.5%). This may be explained by the high average intensity of infection of these two parasites, estimated as 313.8 and 71.45 eggs per gram, respectively. These results are qualitatively similar to some previous studies, with sensitivity of PCR and KK at 98% and 70%, respectively, for *As. lumbricoides* [30] and 94% and 62% for *O. viverrini* [51]. On the other hand, although hookworm prevalence was moderate in Tak (11–15%), neither of the two diagnostics seemed to perform particularly well, with average sensitivity below 45%, which contrasts with previously reported sensitivity for PCR of 98%, while 32% was previously reported for KK [30]. Although many studies found the detection of hook-worm can be improved by using the multiplex qPCR [26,27,62], our model was unable to accurately estimate the sensitivity of either of these two diagnostics, with very wide credible intervals calculated. The efficacy of the qPCR testing could be influenced by suboptimal methods of sample storage and transportation from the field. This is a common challenge faced by research in low or limited resource settings. To be more precise, a limitation of multiplex qPCR detection in our study was the definite detection of only one human species, *N. americanus*. The primers used could not differentiate between *An. duodenale* (in human) and *Ancylostoma ceylanicum* (in zoonoses) under *Ancylostoma* spp. [23]. In addition, neither KK nor the multiplex qPCR performed well in the detection of *Tr. trichiura* in our study. For multiplex qPCR, this could be explained by the thickness of *Tr. trichiura* egg-shell, resulting in incomplete cell lysis and DNA extraction [63]. We were again unable to accurately estimate sensitivity with these two diagnostics. Our findings are in agreement with data reported in a previous meta-analysis for STH, especially for low-intensity transmission settings and hookworm [64], as well as current work in the Philippines (Paller *et al*., unpublished data) [23,26,27,62,63].

The strategic plan of the WHO aims to control and reduce morbidity by eliminating heavy infections, and interrupt transmission [1,65]. Many countries are moving from morbidity control programmes towards transmission elimination, as a result of decreased disease burden owing to preventive chemotherapy. Different levels of control (breaking transmission, pre-elimination or post-elimination) will require different minimal sensitivity levels from the diagnostic techniques used for decision-making. The KK method has been used extensively to screen, evaluate and/or monitor helminth status in endemic countries [13,14,16]. However, variation in sensitivity, technician expertise and difficulties in morphologically identifying the parasites are the main limitations [61]. In the pre-elimination stage, during the evaluation of mass drug administration (MDA) treatment or post-MDA surveillance, understanding the performance (e.g. accuracy and/or sensitivity) of the diagnostic methods employed is important [61,66]. Reduction in the number of helminths in hosts decreases the egg output, which affects the sensitivity of stool examination by the KK method. The multiplex qPCR method was chosen owing to the expected higher sensitivity to complement surveillance and support elimination strategies [67]. However, multiplex qPCR has several limitations including: high cost, specimen processing difficulty, laboratory infrastructure requirements and needing personal with molecular skills, which are all barriers to routinely implementing qPCR into a surveillance system. In addition, it is still unclear on how the outputs (Ct-value) of qPCR relate to the intensity of infection [27].

This study demonstrated that multiplex qPCR can be more sensitive in some settings compared to KK, but KK remains a useful diagnostic tool for common helminthiasis in Thailand. Both diagnostics suffer from similar limitations when prevalence/intensity of infection is low, but KK remains easier to implement in the field. Bayesian LCA is a useful framework to explore different diagnostics, and, for example, guidance for sampling was provided based on the outcomes of such a model in the context of cystic Echinococcosis infections [68]. However, these models can be biased depending on the setting, prevalence and worm burden/infection intensity [69]. Assessing the sensitivity of the diagnostic tools in these diverse settings can be challenging and where data is available, some new techniques may be explored and compared [70]. Future studies seeking to inform planning of surveillance activities should include data on intensity of infection, represented by the number of eggs collected, as well as performing cost-effectiveness analyses to assess optimal diagnostic strategies. Further research is still needed in order to find suitable diagnostics that can perform appropriately at low prevalence levels.

## Supplementary Material

Supplementary file 1

Supplementary file 2

Supplementary file 3

## Figures and Tables

**Table 1:** Advantages and disadvantages of KK and multiplex qPCR technique to detect helminths [16].

attribute	Kato-Katz	multiplex qPCR	sources
skill	detect egg helminth under microscope and requires personal expertise	detect target DNA in stool sample	[16]
cost	USD 1.5	USD 24.2	[23]
time	estimate 20–30 min; single smear 20 min, duplicate smears 27 min	more than 30 min; required DNA extraction step and PCR process	[48]
sample process	short time to process fresh stool samples	long time to process stool with preservative reagent	[16]
contamination	unlikely to contaminate	easy to contaminate	[16]
laboratory infrastructure	equipment and reagent: a light microscope, the Kato-Katz kit (template, slides, spatula, hydrophilic cellophane) and glycerol-malachite green	equipment and reagent: DNA extraction kit, centrifuge, commercial PCR mixer, primer, real-time PCR	[49,50]
reported sensitivity	*As. lumbricoides* (49–70%)	*As. lumbricoides* (79–98%)	[26,30,31,51]
	*Tr. Trichiura* (52–84%)	*Tr. Trichiura* (90–91%)	
	hookworm (32–72%)	hookworm (91–98%)	
	*O. viverrini* (62%)	*O. viverrini* 93.7%	

**Table 2:** Prevalence and sensitivities (95% BCI) for different diagnostic tests on different helminths by province using LCA.

helminth spp.	province	sample size	prevalence reported [23]	Bayesian LCA prevalence (95% BCI)		sensitivity, % (95% BCI)	
				multiplex qPCR	Kato-Katz	multiplex qPCR	Kato-Katz
*O. viverrini*	Tak	167	2.4	2.08 (0.5321–4.659)	2.648 (0.8174–5.49)	22.3 (0.07–92.35)	24.96 (0.139–94.13)
	Ubon Ratchathani	200	28	26.73 (20.89–33.03)	11.56 (7.545–16.22)	88.63 (69.83–99.5)	59.15 (29.14–97.04)
	Sisaket	200	22	21.8 (16,43–27.68)	13.01 (8.767–17.94)	94.19 (81.58–99.79)	73.05 (46.96–98.25)
*As. lumbricoides*	Tak	167	7.78	7.754 (4.234–12.19)	7.714 (4.306–12.15)	88.32 (65.21–99.56)	88.43 (65.3–99.54)
hookworm	Tak	167	Am	11.41 (7.12–16.49)	15.46 (10.38–21.19)	36.14 (0.25–96.42)	42.89 (0.67–97.92)
	Sisaket	200	4	0.026 (0.9–5.24)	4.096 (1.83–7.186)	32.2 (0.06–95.07)	39.1 (0.2–97.43)
*Taenia* spp.	Tak	167	0.65	1.53 (0.29–3.826	0.98 (0.09–2.88)	21.36 (0.09–92.68)	17.99 (0.03–89.48)
	Ubon Ratchathani	200	3.5	3.564 (1.484–6.506)	4.025 (1.779–7.135)	81.24 (47.58–99.29)	86.13 (55.29–99.6)
*Tr. trichiura*	Tak	167	4.79	5.021 (2.282–8.755)	2.13 (0.5604–4.771)	20.4 (0.09–89.52)	28.68 (0.5–94.78)
	Ubon Ratchathani	200	1	0.85 (0.09–2.5)	1.7 (0.46–3.99)	15.41 (0.02–86.86)	20.67 (0.1–92.62)

## Data Availability

All data used in the study were previously shared through the published article and its additional files from the Biomed Central repository: doi:10.1186/s13071-020-04290-0 [23]. The data are provided in the electronic supplementary material [71].
