# Correction: ‘Evaluation of Kato-Katz and multiplex quantitative polymerase chain reaction performance for clinical helminth infections in Thailand using a latent class analysis’ (2023), by Rotejanaprasert *et al.*

**DOI:** 10.1098/rstb.2023.0510

**Published:** 2024-03-25

**Authors:** Chawarat Rotejanaprasert, Pavadee Chuaicharoen, Joaquin M. Prada, Thanawadee Thantithaveewat, Poom Adisakwattana, Wirichada Pan-ngum

**Affiliations:** ^1^ Department of Tropical Hygiene, Mahidol University, Bangkok, Thailand; ^2^ Mahidol-Oxford Tropical Medicine Research Unit (MORU), Mahidol University, Bangkok, Thailand; ^3^ Department of Helminthology, Faculty of Tropical Medicine, Mahidol University, Bangkok, Thailand; ^4^ Faculty of Health and Medical Sciences, School of Veterinary Medicine, University of Surrey, Guildford, UK; ^5^ Bureau of General Communicable Diseases, Ministry of Public Health, Nonthaburi, Thailand

**Keywords:** helminth, elimination, Kato-Katz, qPCR, latent class analysis

*Phil. Trans. R. Soc. B* **378**, 20220281 (Published online 21 August 2023). (https://doi.org/10.1098/rstb.2022.0281)

An error was identified in this paper post-publication. Among the samples examined only one case of *Taenia solium* infection was detected by multiplex qPCR, and therefore could not be used as an example species for analysis. On the other hand, the KK technique cannot identify specific species. Corrections have been made to the Abstract and Introduction to clarify this.

In addition, some errors were found in the data table in the electronic supplementary material, file S3 and have been corrected. These changes do not affect the modelling outputs or conclusions.

This has been corrected on the publisher's website.

