# Supplementary material for: Evaluation of Kato-Katz and multiplex quantitative polymerase chain reaction performance for clinical helminth infections in Thailand using a latent class analysis
Source: Philos Trans R Soc Lond B Biol Sci. Author manuscript; Available in PMC 2023 Oct 9. (PMC10440171; doi:10.1098/rstb.2022.0281)
Supplement: Supplementary file 1 [file EMS185003-supplement-Supplementary_file_1.docx]

**Supplementary file 1**

**Text S1: Latent class modeling for helminth diagnostic tests**

Let *y*_ij_ be the binary observation (0=Negative, 1=Positive) from the *j*th test (*T*_ij_) on the *i*th subject for each helminth Species and area with true infection status, *D*_i_, (0=No, 1=Yes), where *i*=1,…,*N* and *j* = 1,…,*J*. Here *J* = 2 where *j*=1 refers to the Kato-Katz and *j*=2 is qPCR. The results for each individual test follow a Bernoulli distribution with *P*(*Y*_ij_=1|*D*_i_=*d*_i_) which is the probability of testing positive on the *j*th test given an individual’s true helminthic status *d*_i_. The conditional independence assumption can be expressed as

(*Y*_i1_= *y*_1_, *Y*_i2_= *y*_2_ | *D*_i_=*d*_i_) = П_j_ *P*(*Y*_ij_= *y*_j_ | *D*_i_=*d*_i_) (1)

This can be expressed in terms of the test Sensitivities and Specificities as

*P*(*Y*_i1_= *y*_1_, *Y*_i2_= *y*_2_ | *D*_i_=1) = П_j_ (*Se_j_*)^yj^ (1- *Se_j_*)^(1-yj)^

and *P*(*Y*_i1_= *y*_1_, *Y*_i2_= *y*_2_ | *D*_i_=0) = П_j_ (*Sp_j_*)^(1-yj)^ (1- *Sp_j_*)^yj^ (2)

where *Se_j_* = *P*(*Y*_ij_=1 | *D*_i_=1) = *P*(*Y*_j_=1 | *D*_i_=1) being the Sensitivity and *Sp_j_* = *P*(*Y*_ij_=0 | *D*_i_=0) = *P*(*Y*_j_=0 | *D*_i_=0) being the specificity of the *j*th test. The test outcomes can then be modeled using the class probabilities

*P*(*Y*_i1_= *y*_1_, *Y*_i2_= *y*_2_)

= *P*(*D*_i_=0) П_j_ *P*(*Y*_ij_= *y*_j_ | *D*_i_=0) + *P*(*D*_i_=1) П_j_ *P*(*Y*_ij_= *y*_j_ | *D*_i_=1)

= π П_j_ (*Se_j_*)^yj^ (1- *Se_j_*)^(1-yj)^ + (1-π) П_j_ (*Sp_j_*)^(1-yj)^ (1- *Sp_j_*)^yj^ (3)

where π is the prevalence of each helminth in a particular area. The number of class probabilities = 2^J^ and the vector of the class probabilities is denoted by ***P***_y_. Let the number of subjects in each class outcome of *Y*_1_= *y*_1_, *Y*_2_= *y*_2_ be *n*_y1y2_. Then the subject counts follow a multinomial distribution with the class probability defined above, i.e., ***n***_y1y2_ ~ Multinomial (N, ***P***_y_). Moreover, positive (PPV) and negative (NPV) predictive values for the *j*th test for a particular helminth in each area can be expressed as

*PPV*_j_ = π(*Se_j_*)/(π(*Se_j_*)+(1- π)(1- *Se_j_*))

and *NPV*_j_ = (1-π)(*Sp_j_*)/(π(1- *Se_j_*)+(1- π)(*Sp_j_*)). (4)

Since we enter an elimination period in Thailand, the amount of observed data is not large though there are still some scattered hot spots. Frequentist methods such as maximum likelihood can be used to fit; however the Bayesian framework is preferable (1). The prior distribution of sensitivity, specificity and prevalence for each helminthic species was assumed to follow a non-informative distribution as *Beta* (1,1).

**Text S2: OpenBUGS code for the likelihood and test characteristics of latent class modeling for helminth diagnostics**

x[1:4] ~ dmulti(p[1:4], n)

p[1] <- pi*Se[1]*Se[2] + (1-pi)*(1-Sp[1]) *(1-Sp[2])

p[2] <- pi*Se[1]*(1-Se[2])+ (1-pi)*(1-Sp[1])*Sp[2]

p[3] <- pi*(1-Se[1])*Se[2] + (1-pi)*Sp[1]*(1-Sp[2])

p[4] <- pi*(1-Se[1])*(1-Se[2])+ (1-pi)*Sp[1]*Sp[2]

ppv[1]<-pi*Se[1]/(pi*Se[1]+(1-pi)*(1-Sp[1]))

npv[1]<-(1-pi)*Sp[1]/((1-pi)*Sp[1]+pi*(1-Se[1]))

ppv[2]<-pi*Se[2]/(pi*Se[2]+(1-pi)*(1-Sp[2]))

npv[2]<-(1-pi)*Sp[2]/((1-pi)*Sp[2]+pi*(1-Se[2]))

#Prior

pi ~ dbeta(1,1)

Se[1] ~ dbeta(1,1)

Sp[1] ~ dbeta(1,1)

Se[2] ~ dbeta(1,1)

Sp[2] ~ dbeta(1,1)

**Reference**

1. Mweu MM, Wambua J, Njuga F, Bejon P, Mwanga D. Bayesian evaluation of the performance of three diagnostic tests for Plasmodium falciparum infection in a low-transmission setting in Kilifi County, Kenya. Wellcome open research. 2019;4.
