# Supplementary material for: Evaluation of Kato-Katz and multiplex quantitative polymerase chain reaction performance for clinical helminth infections in Thailand using a latent class analysis
Source: Philos Trans R Soc Lond B Biol Sci. Author manuscript; Available in PMC 2023 Oct 9. (PMC10440171; doi:10.1098/rstb.2022.0281)
Supplement: Supplementary file 2 [file EMS185003-supplement-Supplementary_file_2.docx]

**Supplementary file**

**Table S1: Estimates of performance metrics of the three diagnostic tests for Helminths using Latent Class Analysis (LCA)**

***O. viverrini***

| **Tak** | qPCR | Kato-Katz |
| --- | --- | --- |
| Sensitivity | 22.30 (0.07-92.35) | 24.96(0.14-94.13) |
| Specificity | 73.94(6.25-99.91) | 71.11(5.21-99.85) |
| PPV | 49.75(3.48-96.40) | 50.28(3.53-96.41) |
| NPV | 45.83(0.47-99.94) | 45.82(0.45-99.95) |
| *prev* | 2.08(0.53-4.66) | 2.65(0.82-5.49) |
| **Ubon Ratchathani** |  |  |
| Sensitivity | 88.63(69.83-99.50) | 59.15(29.14-97.04) |
| Sp | 87.949(77.41-99.15) | 98.24(95.04-99.91) |
| PPV | 62.18(28.83-97.68) | 87.51(66.03-99.38) |
| NPV | 96.939(89.73-99.89) | 90.11(75.69-99.63) |
| *prev* | 26.73(20.89-33.03) | 11.56(7.55-16.22) |
| **Sisaket** |  |  |
| Sensitivity | 94.19(81.58-99.79) | 73.05(46.96-98.25) |
| Specificity | 93.37(86.02-99.60) | 99.03(96.90-99.97) |
| PPV | 74.38(46.93-98.57) | 93.83(80.62-99.78) |
| NPV | 98.70(95.55-99.96) | 94.22(86.06-99.77) |
| *prev* | 21.80(16.43-27.68) | 13.01(8.77-17.94) |

***A. lumbricoides***

| **Tak** | qPCR | Kato-Katz |
| --- | --- | --- |
| Sensitivity | 88.32(65.21-99.56) | 88.43(65.30-99.54) |
| Specificity | 99.02(96.89-99.97) | 99.04(96.87-99.97) |
| PPV | 88.33(65.47-99.59) | 88.50(65.29-99.59) |
| NPV | 98.99(96.66-99.97) | 99.00(96.61-99.97) |
| *prev* | 7.75(4.23-12.19) | 7.71(4.30-12.15) |

**Hookworms**

| **Tak** | qPCR | Kato-Katz |
| --- | --- | --- |
| Sensitivity | 36.14(0.25-96.42) | 42.89(0.67-97.92) |
| Specificity | 63.80(3.84-99.73) | 57.03(1.86-99.37) |
| PPV | 50.07(1.99-97.88) | 49.90(3.65-96.71) |
| NPV | 49.98(0.39-99.63) | 49.99(0.23-99.74) |
| *prev* | 11.41(7.12-16.49) | 15.46(10.38-21.19) |
| **Sisaket** |  |  |
| Sensitivity | 32.20(0.06-95.07) | 39.10(0.20-97.43) |
| Specificity | 73.42(5.48-99.95) | 67.51(2.90-99.82) |
| PPV | 52.37(2.97-97.65) | 50.91(5.64-95.59) |
| NPV | 55.28(0.01-99.92) | 55.34(0.06-99.94) |
| *prev* | 2.60(0.09-5.24) | 4.10(1.83-7.19) |

***Taenia* spp.**

| **Tak** | qPCR | Kato-Katz |
| --- | --- | --- |
| Sensitivity | 21.36(0.09-92.68) | 17.99(0.03-89.48) |
| Specificity | 80.23(8.15-99.91) | 83.43(11.80-99.97) |
| PPV | 49.32(0.99-98.80) | 49.43(1.55-98.50) |
| NPV | 52.15(0.02-99.99) | 52.14(0.02-99.98) |
| *prev* | 1.53(0.29-3.83) | 0.98(0.09-2.88) |
| **Ubon Ratchathani** | | |
| Sensitivity | 81.24(47.58-99.29) | 86.13(55.29-99.60) |
| Specificity | 99.48(98.11-99.90) | 99.21(97.46-99.97) |
| PPV | 85.96(54.02-99.57) | 81.06(47.57-99.24) |
| NPV | 99.22(97.24-99.98) | 99.43(97.99-99.99) |
| *prev* | 3.56(1.48-6.51) | 4.03(1.78-7.14) |

***T. trichiura***

| **Tak** | qPCR | Kato-Katz |
| --- | --- | --- |
| Sensitivity | 20.40(0.09-89.52) | 28.68(0.50-94.78) |
| Specificity | 82.31(12.11-99.92) | 74.11(5.65-99.40) |
| PPV | 50.47(3.16-96.89) | 49.05(2.47-97.33) |
| NPV | 53.06(0.08-99.20) | 53.07(0.07-99.94) |
| *prev* | 5.02(2.28-8.76) | 2.13(0.56-4.77) |
| **Ubon Ratchathani** | | |
| Sensitivity | 15.41(0.02-86.86) | 20.67(0.01-92.62) |
| Specificity | 84.22(0.52-99.97) | 78.84(7.26-99.86) |
| PPV | 50.08(1.63-98.38) | 50.34(0.61-99.05) |
| NPV | 49.43(0.02-99.98) | 49.43(0.02-99.98) |
| *prev* | 0.85(0.09-2.50) | 1.70(0.46-3.99) |
