# Supplementary material for: Evaluation of Kato-Katz and multiplex quantitative polymerase chain reaction performance for clinical helminth infections in Thailand using a latent class analysis
Source: Philos Trans R Soc Lond B Biol Sci. Author manuscript; Available in PMC 2023 Oct 9. (PMC10440171; doi:10.1098/rstb.2022.0281)
Supplement: Supplementary file 3 [file EMS185003-supplement-Supplementary_file_3.docx]

**Supplementary file 3**

Based on the study by Adisakwattana et. al. (2020) [1], 567 fecal samples were collected between December 2017 and February 2018, and analyzed by KK and multiplex qPCR analysis. The result is given in Table S1

**Table S1: Laboratory test results by Kato-Katz and multiplex qPCR**

| Helminth spp. | Study sites |  | Kato-Katz positive | Kato-Katz negative | Total |
| --- | --- | --- | --- | --- | --- |
| 1. *O. viverrini* | Tak | qPCR positive | 2 | 0 | 2 |
|  |  | qPCR negative | 1 | 164 | 165 |
|  |  | Total | 3 | 164 | 167 |
|  | Ubon Ratchathani | qPCR positive | 19 | 34 | 53 |
|  |  | qPCR negative | 3 | 144 | 147 |
|  |  | Total | 22 | 178 | 200 |
|  | Sisaket | qPCR positive | 24 | 19 | 43 |
|  |  | qPCR negative | 1 | 156 | 157 |
|  |  | Total | 25 | 175 | 200 |
| 2. *A. lumbricoides* | Tak | qPCR positive | 11 | 1 | 12 |
|  |  | qPCR negative | 1 | 154 | 155 |
|  |  | Total | 12 | 155 | 167 |
| 3. Hookworm | Tak | qPCR positive | 12 | 5 | 17 |
|  |  | qPCR negative | 12 | 137 | 150 |
|  |  | Total | 25 | 142 | 167 |
|  | Ubon Ratchathani | qPCR positive | 0 | 0 | 0 |
|  |  | qPCR negative | 1 | 199 | 200 |
|  |  | Total | 1 | 199 | 200 |
|  | Sisaket | qPCR positive | 3 | 1 | 4 |
|  |  | qPCR negative | 4 | 192 | 196 |
|  |  | Total | 7 | 193 | 200 |
| 4. *Taenia* spp. | Tak | qPCR positive | 0 | 1 | 1 |
|  |  | qPCR negative | 0 | 166 | 166 |
|  |  | Total | 0 | 167 | 167 |
|  | Ubon Ratchathani | qPCR positive | 6 | 0 | 6 |
|  |  | qPCR negative | 1 | 193 | 194 |
|  |  | Total | 7 | 193 | 200 |
| 5. *T. Trichiura* | Tak | qPCR positive | 1 | 1 | 2 |
|  |  | qPCR negative | 6 | 159 | 165 |
|  |  | Total | 7 | 160 | 167 |
|  | Ubon Ratchathani | qPCR positive | 0 | 0 | 0 |
|  |  | qPCR negative | 2 | 198 | 200 |
|  |  | Total | 2 | 198 | 200 |


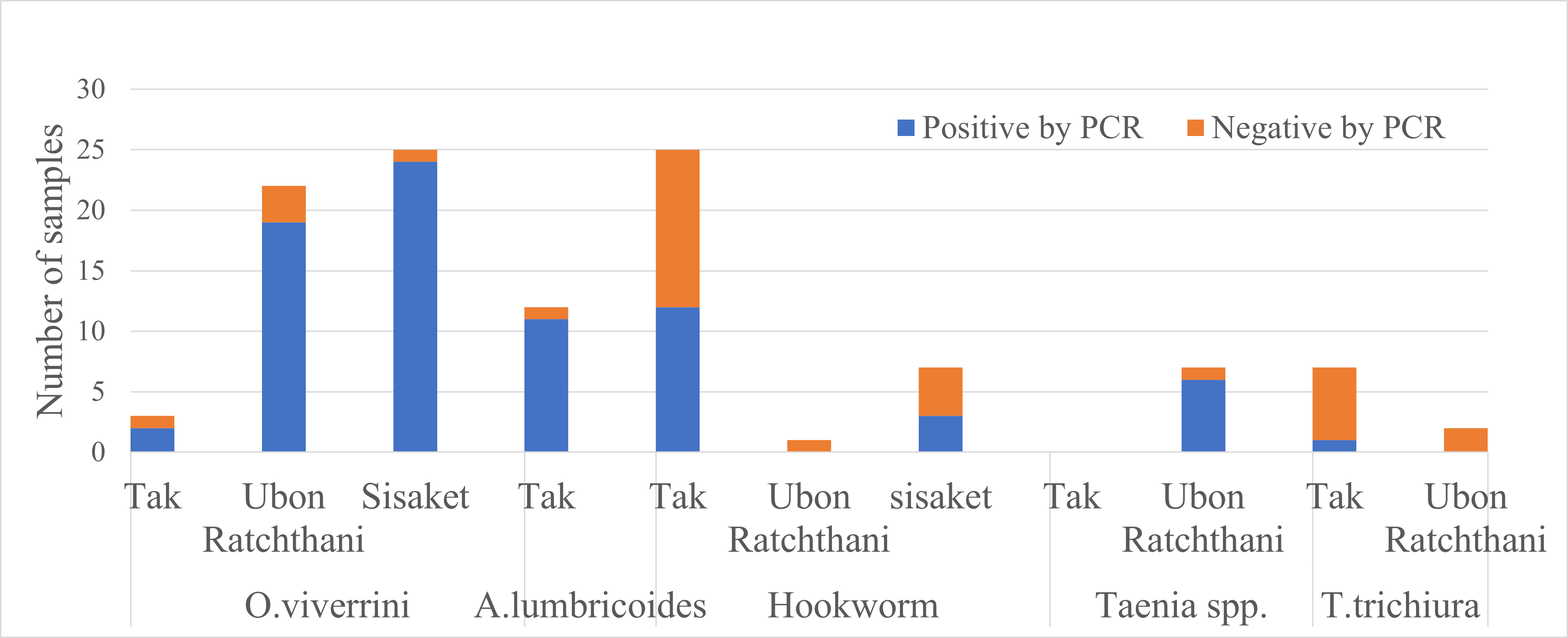


**Figure S1: The number of positive and negative multiplex qPCR sample given the Kato-Katz result was positive.**

The table and figure confirmed the expected observations where the test agreement is specific to species and number of samples i.e. lower agreement in Hookworm and when number of samples is small.

On the other hand, given a positive PCR sample, the number of positive and negative KK varied with species and settings.


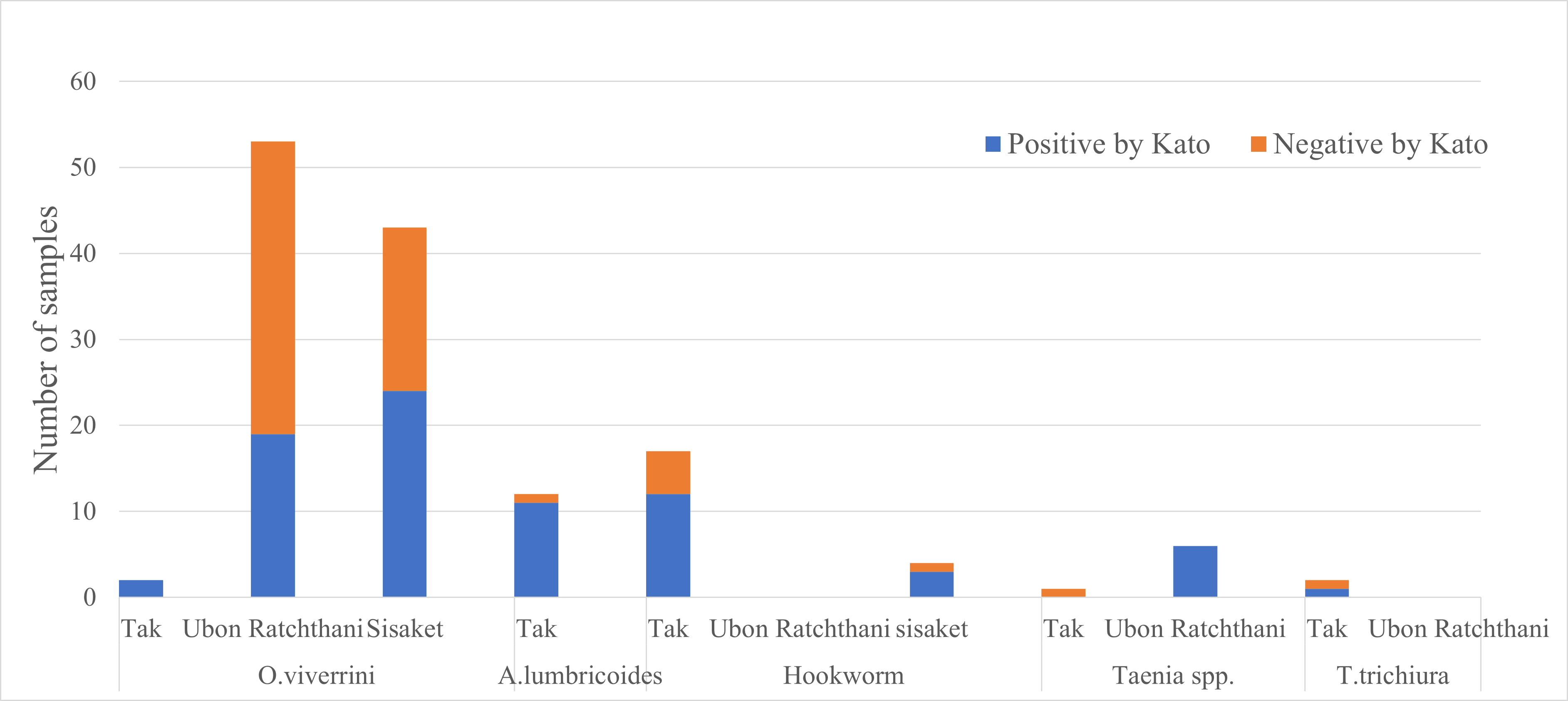


**Figure S2: The number of positive and negative Kato-Katz sample given the multiplex qPCR result was positive.**

**References**

1. Adisakwattana P, Yoonuan T, Phuphisut O, Poodeepiyasawat A, Homsuwan N, Gordon CA, et al. Clinical helminthiases in Thailand border regions show elevated prevalence levels using qPCR diagnostics combined with traditional microscopic methods. Parasites & Vectors. 2020;13(1). doi: ARTN 416

10.1186/s13071-020-04290-0. PubMed PMID: WOS:000562803800004.
